# Effectiveness of Insulin Versus Oral Agents in Patients with Uncontrolled Type 2 Diabetes Mellitus: A Retrospective Comparative Study

**DOI:** 10.7759/cureus.90196

**Published:** 2025-08-15

**Authors:** Muhammad Hamza Naeem, Mudassir Raza, Moaz Bin Aziz, Najeeb Ullah, Abdul Wahab, Zoya Azhar Khan, Hinza Farooq

**Affiliations:** 1 Internal Medicine, Punjab Medical College, Faisalabad, PAK; 2 Internal Medicine, Sheikh Zayed Hospital, Rahim Yar Khan, PAK; 3 Internal Medicine, Services Hospital, Lahore, PAK; 4 Internal Medicine, Fatima Memorial Hospital, Lahore, PAK; 5 Internal Medicine, Faisal Hospital, Faisalabad, PAK; 6 Internal Medicine, Shalamar Hospital, Lahore, PAK

**Keywords:** blood glucose, diabetes mellitus, glycated hemoglobin (hba1c), hypoglycemic agents, insulin, type 2 diabetes mellitus

## Abstract

Background

Type 2 diabetes mellitus (T2DM) is a chronic, progressive metabolic disorder characterized by insulin resistance and declining beta-cell function, resulting in elevated blood glucose levels. Its multifactorial pathogenesis involves complex interactions between genetic predisposition and environmental factors such as obesity, sedentary lifestyle, and diet. While lifestyle modifications remain the cornerstone of management, pharmacological intervention is often required to achieve and maintain optimal glycemic control.

Objective

This study aimed to evaluate the effectiveness of insulin therapy versus oral hypoglycemic agents in controlling blood glucose, along with assessing the safety profiles and patient adherence to both treatment modalities.

Methods

This retrospective study was conducted at Services Hospital, Lahore, Pakistan, from December 2023 to December 2024. A total of 255 patients diagnosed with uncontrolled T2DM were included in the study. Data were collected through medical records by using a structured proforma, which included demographic details (age, gender, BMI), medical history (duration of diabetes, comorbidities), current medication regimen (insulin or oral agents), and laboratory measurements (HbA1c, fasting blood glucose). Other clinical parameters, such as blood pressure, weight, and presence of complications (e.g., neuropathy, retinopathy), were also recorded.

Results

Insulin therapy resulted in a greater reduction in HbA1c (mean change -1.3%) and fasting blood glucose (mean change -38.2 mg/dL) compared to oral agents (mean change in HbA1c -0.6%, mean change in fasting blood glucose -25.6 mg/dL), with both groups showing statistically significant improvements (p < 0.001). However, insulin therapy was associated with a higher incidence of hypoglycemia (18% vs. 7%, p = 0.02) and weight gain (mean increase 2.3 ± 1.4 kg vs. 0.9 ± 0.8 kg, p < 0.001). Oral agents were linked to more gastrointestinal side effects (15% vs. 5%, p = 0.01). Patient adherence was similar between the two groups, with no significant difference (90% in the oral agents group vs. 85% in the insulin therapy group).

Conclusion

Insulin therapy offers superior glycemic control compared to oral agents but carries a higher risk of hypoglycemia and weight gain. Oral agents, while less effective, have a safer side effect profile and are preferred in patients at risk of hypoglycemia or those concerned about weight gain. Treatment choice should be individualized, considering patient preferences, comorbidities, and the risks and benefits of each therapy.

## Introduction

Diabetes mellitus (DM), particularly type 2 diabetes mellitus (T2DM), is a global health concern, with increasing prevalence rates worldwide. The condition is characterized by insulin resistance and beta-cell dysfunction, leading to chronic hyperglycemia and associated complications such as cardiovascular diseases, nephropathy, neuropathy, and retinopathy [1]. Although a lifestyle change is the basis of controlling diabetes, the use of pharmacological treatment becomes imperative when the body loses its control over glycemic levels using diet and physical activities [2]. There are mainly insulin therapy and different oral hypoglycemic agents with their unique actions, advantages, and drawbacks. Insulin, a key anabolic hormone that facilitates cellular glucose uptake and inhibits hepatic glucose production, is typically initiated in T2DM patients when progressive pancreatic beta-cell dysfunction leads to insufficient endogenous insulin secretion and oral agents alone fail to achieve adequate glycemic control. It is also the primary therapy in type 1 diabetes mellitus (T1DM) due to absolute insulin deficiency [3]. It is also used exogenously to reduce the level of blood glucose in a direct way, therefore treating the insulin deficiency or resistance cases in patients [4]. Nevertheless, there are such difficulties related to insulin therapy as low blood sugar and weight gain, as well as injection problems. Conversely, oral drugs, such as metformin, sulfonylureas, DPP-4 inhibitors, and SGLT-2 inhibitors, provide the second alternative for people who can still produce insulin inefficiently [5]. These drugs have numerous actions, which include insulin sensitizing, insulin secretion, and glucose reabsorption blockade. Oral agents are often preferred initially due to their convenience and ease of use. However, the risk of hypoglycemia varies among different oral medications; for example, sulfonylureas and meglitinides are associated with a higher risk of hypoglycemia, whereas agents like metformin, DPP-4 inhibitors, and SGLT-2 inhibitors have a lower hypoglycemic risk. Nevertheless, although the use of oral agents is widespread, it is possible to come across situations where the oral agents do not offer sufficient glycemic control and the transition to the use of insulin is required [6]. It brings serious question marks about the effectiveness, safety, and indeed other advantages of insulin as against oral agents among patients with uncontrolled diabetes [7]. This retrospective comparative study aims to evaluate the efficacy and safety of insulin therapy versus oral hypoglycemic agents in achieving optimal glycemic control among patients with T2DM, with additional focus on patient adherence and treatment-related adverse effects [8]. Decision-making for insulin and oral agents should consider many individual patient factors such as disease severity, comorbidity, and patient preference [9]. The therapy choice may be affected by some factors that include the existence of obesity, cardiovascular condition, kidney dysfunction, or a background of hypoglycemic events [10]. While certain oral agents like SGLT-2 inhibitors may benefit patients with obesity, and others, such as metformin, require caution in renal impairment, this study primarily focuses on comparing overall effectiveness and safety profiles of insulin versus oral therapies in uncontrolled T2DM [11]. Insulin therapy limits have been seen in the process of controlling blood glucose. It also usually needs several shots in a single day or the use of an insulin pump, and the danger of hypoglycemia is escalated in case the doses are not properly modified [12].

Objective

This study aimed to evaluate the effectiveness of insulin therapy versus oral hypoglycemic agents in controlling blood glucose, along with assessing the safety profiles and patient adherence to both treatment modalities.

## Materials and methods

Methodology

This retrospective study was conducted at Services Hospital, Lahore, Pakistan, from December 2023 to December 2024. A total of 255 patients diagnosed with uncontrolled T2DM were included in the study. These patients were either on insulin therapy or oral hypoglycemic agents but had suboptimal glycemic control, as evidenced by HbA1c levels greater than 7.0%. A non-probability consecutive sampling method was used to recruit participants.

Inclusion and exclusion criteria

The study included adult patients aged between 40 and 75 years who had been diagnosed with T2DM for at least one year. Eligible participants had an HbA1c level greater than 7.0% but less than or equal to 14.0% despite lifestyle modifications or pharmacological treatment and were either on insulin therapy or oral hypoglycemic agents such as metformin, sulfonylureas, SGLT-2 inhibitors, DPP-4 inhibitors, or GLP-1 receptor agonists. Patients with an HbA1c level exceeding 14.0% were excluded. This upper limit targeted individuals with extremely uncontrolled diabetes, whose distinct clinical trajectories or need for urgent intensive management could disproportionately influence the study outcomes. Patients were excluded if they had T1DM, were pregnant or breastfeeding, or had severe comorbidities such as end-stage renal disease, active cancer, or terminal illness. Additionally, patients with a known hypersensitivity or contraindication to insulin or oral antidiabetic agents, or those with a history of diabetic ketoacidosis or hyperosmolar hyperglycemic state, were also excluded from the study.

Data collection

Data were collected through medical records by using a structured proforma, which included demographic details (age, gender, BMI), medical history (duration of diabetes, comorbidities), current medication regimen (insulin or oral agents), and laboratory measurements (HbA1c, fasting blood glucose). Other clinical parameters, such as blood pressure, weight, and presence of complications (e.g., neuropathy, retinopathy), were also recorded. The major objective of the research was to determine how effective insulin therapy was as compared to the oral agents in the realization of glycemic control. This was quantified by the comparison of the level of HbA1c and the fasting plasma glucose at baseline with those recorded after a three-month follow-up period. Moreover, the number of documented or symptomatic hypoglycemia cases in the study period was also recorded. Hypoglycemia episodes were defined according to the American Diabetes Association criteria [13] as any documented plasma glucose level ≤70 mg/dL accompanied by typical symptoms (e.g., sweating, shakiness, confusion), or any symptomatic event reported by the patient consistent with hypoglycemia, regardless of glucose measurement. The secondary result of the research was aimed at the safety profile of insulin versus the oral agents. This was evaluated through an evaluation of the side effects, which include weight gain, low blood sugar, gastrointestinal disorders (like nausea, vomiting, and diarrhea), and other side effects associated with every treatment.

Statistical analysis

The data were analyzed using IBM SPSS Statistics software, version 26 (IBM Corp., Armonk, NY). Descriptive statistics, including means, standard deviations, frequencies, and percentages, were used to summarize the baseline characteristics and clinical parameters of the study participants. To compare the effectiveness of insulin therapy versus oral agents, changes in HbA1c and fasting blood glucose from baseline to three months were analyzed using analysis of covariance (ANCOVA), adjusting for baseline values of each parameter. This approach accounts for baseline differences and reduces variability. The assumptions for ANCOVA were checked and met. A p-value of < 0.05 was considered statistically significant. The efficacy of insulin versus oral agents was compared by performing chi-square tests for categorical variables like the incidence of hypoglycemia and adverse events. A p-value of less than 0.05 was considered statistically significant.

## Results

The baseline demographics of participants in both the insulin therapy group (127 patients) and the oral agents group (128 patients) were similar. The mean age was 58.4 ± 9.6 years for the insulin therapy group and 58.3 ± 9.8 years for the oral agents group, showing no significant difference. The gender distribution was also comparable, with 62% of males in the insulin therapy group and 58% in the oral agents group. The mean BMI was slightly higher in the insulin therapy group (30.2 ± 4.9 kg/m²) compared to the oral agents group (29.4 ± 4.4 kg/m²), but this difference was not statistically significant. The duration of diabetes was slightly longer in the insulin therapy group (10.5 ± 4.5 years) compared to the oral agents group (9.9 ± 4.1 years), but the difference was minimal (Table 1).

**Table 1:** Baseline demographics of the study participants (n = 255) Data are presented as mean ± SD or n (%). Independent t-test for continuous variables, chi-square (χ²) test for categorical variables; Significance set at p < 0.05.

Characteristic	Insulin therapy group (n = 127)	Oral agents group (n = 128)	Test statistic	p-value
Age (years)	58.5 ± 9.4	58.3 ± 9.8	t = 0.16	0.87
Gender			χ² = 0.66	0.42
Male	79 (62.2%)	74 (57.8%)		
Female	48 (37.8%)	54 (42.2%)		
BMI (kg/m²)	30.2 ± 4.9	29.4 ± 4.4	t = 1.45	0.15
Duration of diabetes (years)	10.5 ± 4.5	9.9 ± 4.1	t = 1.09	0.28
Hypertension	74 (58.3%)	67 (52.3%)	χ² = 0.99	0.32
Dyslipidemia	62 (48.8%)	58 (45.3%)	χ² = 0.64	0.42

After adjusting for baseline values using ANCOVA, insulin therapy was associated with a significantly greater reduction in HbA1c compared to oral agents. The adjusted mean difference in HbA1c change was -0.7% (95% CI: -0.9 to -0.5), with a large effect size (Cohen's d = 0.93), F(1, 252) = 55.3, p < 0.001. Similarly, the adjusted mean difference in fasting blood glucose change was -12.6 mg/dL (95% CI: -16.2 to -8.9), with a moderate effect size (Cohen's d = 0.71), F(1, 252) = 32.1, p < 0.001 (Table 2).

**Table 2:** Comparison of glycemic control (HbA1c and fasting blood glucose) at baseline and after three months of treatment Data are presented as mean ± SD. Adjusted mean differences and F-statistics are from analysis of covariance (ANCOVA), adjusting for baseline values. A p-value of < 0.05 is considered statistically significant.

Parameter	Insulin therapy group (n=127)	Oral agents group (n=128)	Adjusted mean difference (95% CI)	F-statistic	p-value
HbA1c at baseline (%)	8.5 ± 1.3	8.4 ± 1.3	—	—	—
HbA1c after three months (%)	7.2 ± 1.1	7.8 ± 1.2	—	—	—
Mean change in HbA1c (%)	-1.3 ± 0.8	-0.6 ± 0.7	-0.7 (-0.9, -0.5)	55.3	<0.001
Fasting blood glucose at baseline (mg/dL)	179.5 ± 25.2	178.2 ± 27.1	—	—	—
Fasting blood glucose after three months (mg/dL)	141.3 ± 22.3	152.6 ± 26.8	—	—	—
Mean change in fasting blood glucose (mg/dL)	-38.2 ± 16.5	-25.6 ± 18.9	-12.6 (-16.2, -8.9)	32.1	<0.001

Adverse events were more common in the insulin therapy group, particularly with hypoglycemia and weight gain. The incidence of hypoglycemia was 18% in the insulin therapy group, compared to 7% in the oral agents group (p-value = 0.02). The insulin therapy group also had a significantly higher rate of weight gain, with 22% of patients gaining an average of 2.3 ± 1.4 kg, compared to 8% of patients in the oral agents group, who gained only 0.9 ± 0.8 kg (p-value < 0.001). Gastrointestinal side effects were more common in the oral agents group (15%) than in the insulin therapy group (5%) (p-value = 0.01) (Table 3).

**Table 3:** Adverse events and weight change in both treatment groups Data are presented as mean ± SD or n (%). Independent samples t-test for weight gain; chi-square (χ²) test for all other categorical comparisons. Significance set at p < 0.05.

Adverse event/Parameter	Insulin therapy group (n = 127)	Oral agents group (n = 128)	Test statistic	p-value
Hypoglycemia	23 (18.1%)	9 (7.0%)	χ² = 7.13	0.02
Weight gain	28 (22.0%)	10 (7.8%)	χ² = 10.96	< 0.001
Mean weight gain (kg)	2.3 ± 1.4	0.9 ± 0.8	t = 8.29	< 0.001
Gastrointestinal side effects	6 (4.7%)	19 (14.8%)	χ² = 6.56	0.01

The prevalence of pre-existing comorbidities, including hypertension, dyslipidemia, neuropathy, retinopathy, and chronic kidney disease, was similar between the insulin and oral agents groups, with no statistically significant differences observed (p-values ranged from 0.32 to 0.76). This indicates that the two treatment groups had comparable rates of diabetes-related complications and comorbidities at baseline (Table 4).

**Table 4:** Comparison of comorbidities and complications between the two treatment groups Data are presented as n (%). The chi-square (χ²) test is used. Significance level: p < 0.05.

Complication	Insulin therapy group (n = 127)	Oral agents group (n = 128)	χ² Value	p-value
Hypertension	74 (58.3%)	67 (52.3%)	0.99	0.32
Dyslipidemia	62 (48.8%)	58 (45.3%)	0.64	0.42
Neuropathy	23 (18.1%)	26 (20.3%)	0.13	0.72
Retinopathy	15 (11.8%)	13 (10.2%)	0.29	0.59
Chronic kidney disease	11 (8.7%)	10 (7.8%)	0.09	0.76

The patient adherence rate was slightly higher in the oral agents group (90%) compared to the insulin therapy group (85%), although this difference was not statistically significant (p-value = 0.12). Non-adherence was reported by 15% of patients in the insulin therapy group and 10% in the oral agents group, with no significant difference between the groups (p-value = 0.12). The reasons for non-adherence varied: 8% of patients in the insulin therapy group cited hypoglycemia risk, 7% reported injection discomfort, and 3% mentioned forgetfulness (Table 5).

**Table 5:** Patient adherence to treatment in both groups Data are presented as n (%). The chi-square (χ²) test is used. Significance set at p < 0.05.

Adherence measure	Insulin therapy group (n = 127)	Oral agents group (n = 128)	χ² Value	p-value
Adherence rate	108 (85.0%)	115 (89.8%)	2.48	0.12
Self-reported non-adherence	19 (15.0%)	13 (10.2%)	2.48	0.12
Hypoglycemia risk (reason)	10 (7.9%)	—	4.58	0.03
Injection discomfort (reason)	9 (7.1%)	—	6.26	0.01
Forgetfulness	4 (3.1%)	5 (3.9%)	0.05	0.82

## Discussion

The results of this study provide valuable insights into the comparative effectiveness and safety of insulin therapy versus oral hypoglycemic agents in the management of uncontrolled T2DM. The answer to the above main findings is that although insulin therapy resulted in a significantly greater reduction in HbA1c (mean decrease of 1.3% vs. 0.6% with oral agents), it was also associated with a higher incidence of hypoglycemia (18% vs. 7%; absolute risk increase 11%) and greater weight gain (mean increase of 2.3 kg vs. 0.9 kg). These differences highlight the need to weigh the enhanced glycemic control against the increased risk of adverse effects when individualizing treatment. Conversely, oral medication, although somewhat inferior in terms of its effect on blood glucose management, does have a more beneficial side effect, especially in weight control and hypoglycemia risk. The insulin therapy group had a considerably higher improvement in the levels of HbA1c and fasting blood glucose than the oral agents group. The average number of HbA1c decreased among patients treated with insulin by 1.3% versus 0.6% in the oral agents group (p < 0.001). In the same way, the fasting level of blood glucose was reduced by 38.2 mg/dL in the insulin group, with a 25.6 mg/dL decrease in the oral agents group (p < 0.01) [14]. These results are in line with the past studies; Szarek et al. reported that agents enhancing glycemic control in patients with T2DM and heart failure showed modest HbA1c reductions in the range of 0.5% to 1.0%, highlighting that the 1.3% reduction seen with insulin in our study is clinically meaningful [14]. Donner and Sarkar emphasize that insulin directly compensates for beta-cell dysfunction by supplementing endogenous insulin levels, which explains the greater glycemic reductions compared to oral agents that primarily improve insulin sensitivity or secretion [15]. Metformin, the first-line oral agent, primarily reduces hepatic glucose production and improves peripheral insulin sensitivity. While it does not directly replace endogenous insulin, when used in combination with insulin therapy, metformin can lower the required insulin dose and may help mitigate insulin-associated weight gain. This observation is in line with the previous reports, which showed that the risk of hypoglycemia with insulin is higher, especially when the doses were not adjusted correctly and when the patients suffered fluctuations in their daily physical activity and food intake [16].

Weight gain was reported in about 22% of the adopted cluster management options of insulin patients who reported a modest average of 2.3 kg. Conversely, few cases of weight increase (8%) were observed in the oral agents group, and fewer gastrointestinal adverse events, especially with metformin, were reported. Metformin has a weight-neutral or even weight-reducing effect, and this characteristic makes the drug the first line of treatment for many patients with T2DM [17]. Patient adherence rates were relatively high and showed no significant difference between the insulin (85%) and oral agents (90%) groups. However, adherence was assessed based on self-reported data documented in medical records rather than using validated adherence measurement tools. Therefore, these findings should be interpreted cautiously, as self-reporting may overestimate adherence and introduce bias [18]. This is an indication that insulin therapy is an effective treatment alternative for some patients, especially those who demand more rigorous management of their glycemic levels in case of discomfort during injection and hypoglycemia [19]. Nonetheless, the insulin group had a greater rate of hypoglycemia and weight gain, which can influence the adherence to the medication long-term and the quality of life of the patients. The enhanced compliance recorded in the oral agents group can be explained by the fact that it is more convenient to take oral drugs than to be required to make regular insulin injections, and this can be a considerable obstacle to most patients [20]. These challenges for insulin patients are further illustrated by the higher reported reasons of non-adherence in the insulin group, which are the fear of hypoglycemia and the discomfort of injection [21, 22]. The results of the present research indicate that although insulin therapy shows more effectiveness in the management of blood glucose, the emergence of greater risks associated with hypoglycemia and weight gain can reduce its utilization among particular groups of patients, especially those already overweight and with a record of hypoglycemic incidents. Although their efficacy regarding strict glycemic control is lower, oral agents represent a safe alternative, which provides a reduced probability of hypoglycemia and negligible weight gain.

This study has several limitations that should be considered when interpreting the findings. First, the relatively short follow-up period of three months limits the ability to assess long-term glycemic control, safety outcomes, and durability of treatment effects. Second, the use of a non-probability consecutive sampling method may introduce selection bias, affecting the representativeness of the study population. Third, although statistical adjustments were made, the analysis did not fully control for potential confounding variables such as baseline differences in BMI and duration of diabetes, which might have influenced treatment response. Additionally, the study was conducted at a single tertiary care center, which may limit the generalizability of the results to other settings or populations. Finally, adherence was assessed through self-report without validated tools, potentially overestimating compliance.

## Conclusions

This study highlights the comparative effectiveness and safety of insulin therapy versus oral hypoglycemic agents in the management of uncontrolled T2DM. Insulin therapy demonstrated superior efficacy in lowering HbA1c and fasting blood glucose levels compared to oral agents, with an adjusted mean HbA1c reduction of 0.7% greater than oral therapy. However, this improved glycemic control came with an 11% higher absolute risk of hypoglycemia and significantly greater weight gain. Oral agents, while less effective in achieving optimal glycemic control, exhibited a more favorable safety profile.

Given these quantified risks and benefits, treatment choice should be individualized by considering patient-specific factors such as hypoglycemia risk, concerns about weight gain, disease severity, comorbidities, and personal preferences. Insulin therapy may be more suitable for patients requiring more aggressive glycemic control or with advanced beta-cell dysfunction, whereas oral agents may be preferred for those with lower hypoglycemia risk or weight management concerns.

These findings stem from a single-center study with a relatively short follow-up period; therefore, results should be interpreted with caution. Further multicenter studies with longer follow-up are needed to confirm the generalizability and durability of these outcomes.
